# Evaluation of a Novel Rapid Phenotypic Antimicrobial Susceptibility Testing System

**DOI:** 10.3390/antibiotics14100962

**Published:** 2025-09-25

**Authors:** Yuan-Chao Xue, Filipe Cerqueira, Natalie Williams-Bouyer, Ping Ren

**Affiliations:** Department of Pathology, University of Texas Medical Branch, Galveston, TX 77555, USA; yuaxue@utmb.edu (Y.-C.X.); fcerqueira@uabmc.edu (F.C.); nmwillia@utmb.edu (N.W.-B.)

**Keywords:** rapid phenotyping antimicrobial susceptibility testing, Selux DX system, categorical agreement, very major error, major error, minor error

## Abstract

Background/Objectives: Phenotypic antimicrobial susceptibility testing (AST) is essential for guiding timely and effective antibiotic therapy. Rapid and accurate reporting of AST results enables earlier optimization of treatment and supports antimicrobial stewardship by minimizing unnecessary use of broad-spectrum antibiotics. This study aimed to evaluate the performance of the Selux DX Next-Generation Phenotyping AST system in comparison with the standard-of-care MicroScan WalkAway Plus system and broth microdilution reference results. Methods: A total of 332 clinical isolates and 97 Antimicrobial Resistance (AR) Bank reference isolates were tested using the Selux DX and MicroScan systems. Performance was assessed by categorical agreement (CA), error rates [very major errors (VMEs), major errors (MEs), minor errors (mEs)], and turnaround time. Results: The Selux DX system demonstrated ≥90% CA for most drug–organism combinations, consistent with Clinical and Laboratory Standards Institute (CLSI) acceptance thresholds, although elevated error rates were noted for erythromycin, aztreonam, cefazolin, minocycline, and ampicillin/sulbactam. Across 5124 drug–bug combinations, 55 VMEs (1.1%), 42 MEs (0.8%), and 203 mEs (4.0%) were identified. The Selux DX system achieved a markedly shorter average turnaround time of 5.5 h compared with 16 h for the MicroScan system, though at the cost of a longer setup time. Conclusions: The Selux DX system provides rapid and reliable phenotypic AST results, supporting earlier clinical decision-making and antimicrobial stewardship. However, discrepancies with certain antimicrobial agents, particularly among highly resistant reference isolates, highlight the need for further validation in larger, multicenter studies.

## 1. Introduction

The increasing emergence of multidrug-resistant (MDR) bacterial pathogens has significantly reduced the availability of effective therapies, posing a major challenge to the treatment of infectious diseases. Infections caused by MDR organisms, including carbapenem-resistant Enterobacterales (CRE), methicillin-resistant *Staphylococcus aureus* (MRSA), and extended-spectrum β-lactamase (ESBL)-producing bacteria, are associated with prolonged hospital stays, increased morbidity and mortality, and rising healthcare costs [1,2,3,4]. To optimize patient outcomes, rapid and accurate phenotypic antimicrobial susceptibility testing (AST) is essential for guiding targeted antibiotic therapy [5,6,7,8].

However, traditional AST methods, such as broth microdilution and automated susceptibility testing platforms, often require 3–5 days for final result reporting due to the inherent time needed for bacterial growth and culture-dependent processing steps [9,10,11]. This delay necessitates the empiric use of broad-spectrum antibiotics, which not only contributes to increased toxicity and adverse drug reactions but also drives the selection of resistant strains, further exacerbating the global antimicrobial resistance crisis [12,13]. Given these challenges, there is an urgent need for validated and rapid phenotypic AST methods that can deliver accurate and reproducible minimum inhibitory concentration (MIC) data across a broad range of clinically relevant bacterial pathogens and antimicrobial agents.

The Selux DX Next-Generation Phenotyping AST system (Selux DX: Selux Diagnostics Inc., Charlestown, MA, USA) is an innovative, high-throughput, random-access, and fully automated platform designed to provide rapid AST results for both Gram-negative and Gram-positive bacterial isolates. This system received US Food and Drug Administration (FDA) clearance in 2023 [14]. By leveraging the electrostatic binding of a highly fluorescent molecule to bacterial cell surfaces to predict antibiotic susceptibility, Selux DX offers the potential to significantly reduce turnaround time while maintaining accuracy comparable to reference susceptibility testing methods [15].

In this study, we conducted a single-center evaluation of the Selux DX system, comparing its AST performance and time-to-result with the MicroScan WalkAway Plus Microbiology System (MicroScan: Beckman Coulter Inc., Franklin Lakes, NJ, USA), a widely used automated AST platform. The study also supplemented 97 GN reference isolates with well-established susceptibility profiles, which were obtained from the Centers for Disease Control and Prevention (CDC) & US FDA Antimicrobial Resistance Isolate Bank (AR Bank), for comparative analysis. Our findings aim to inform the clinical microbiology community on the potential of Selux DX to accelerate susceptibility testing and support antimicrobial stewardship initiatives.

## 2. Results

**Figure 1:**
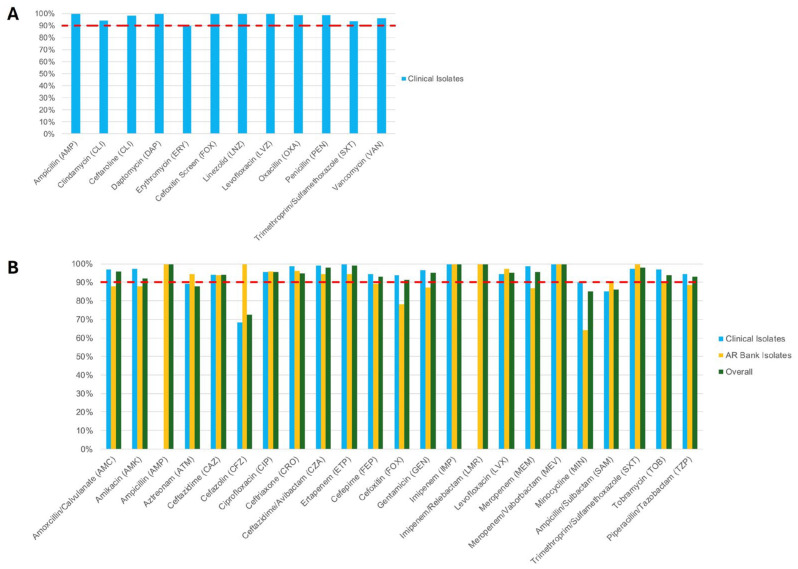
Categorical Agreement (CA) of (**A**) Gram-positive and (**B**) Gram-negative organisms. All values are shown in percentage (%). The red dashed line illustrates CLSI’s 90% CA cut-off.

Among the 109 Gram-positive (GP) clinical isolates, the most common organisms were *S. aureus* (60 isolates, 55.0%)*, Enterococcus faecalis* (24 isolates, 22.0%), and *Staphylococcus epidermidis* (15 isolates, 13.8%) (Table 1). All tested antibiotic-organism combinations for GP isolates demonstrated greater than 90% categorical agreement (CA) except for erythromycin (ERY; 89.5%; 77/86) (Figure 1A and Supplementary Table S1). Among the 223 Gram-negative (GN) clinical isolates, the most frequently encountered were *Escherichia coli* (68 isolates, 30.5%), *Proteus mirabilis* (45 isolates, 20.2%)*, Klebsiella pneumoniae* (38 isolates, 17.0%), and *Pseudomonas aeruginosa* (32 isolates, 14.3%) (Table 1). For these GN clinical isolates, all tested antibiotics exhibited ≥90% CA except for aztreonam (ATM; 89.2%; 83/93), cefazolin (CFZ; 69.0%; 109/158), and ampicillin/sulbactam (SAM; 85.1%; 148/174) (Figure 1B and Supplementary Table S2). In the evaluation of 97 GN AR Bank isolates, most antibiotics also achieved ≥90% CA, with the exception of amoxicillin (AMC; 88.0%; 22/25), amikacin (AMK; 88.0%; 44/50), ATM (84.4%; 27/32), cefepime (FEP; 89.4%; 59/66), cefoxitin (FOX; 78.1%; 25/32), gentamicin (GEN; 87.2%; 34/39), meropenem (MEM; 86.9%; 73/84), minocycline (MIN; 64.3%; 18/28), and piperacillin/tazobactam (TZP; 88.7% 63/71) (Figure 1B and Supplementary Table S2). When combining the clinical and AR Bank GN isolates (total *n* = 320), ≥90% CA was observed for most drug–organism combinations. Notable exceptions included ATM (88.0%; 110/125), CFZ (73.1%; 133/182), MIN (85.1%; 126/148), and SAM (86.2%; 194/225).

**Figure 2:**
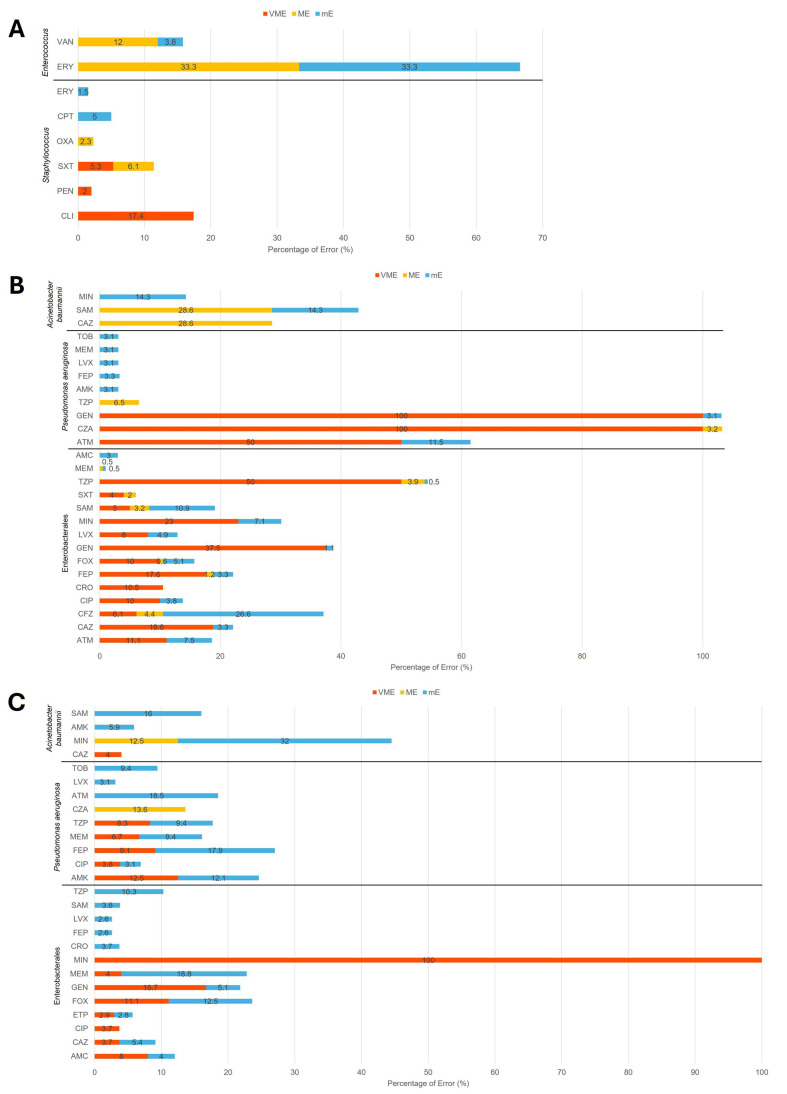
Category Discrepancies of (**A**) Gram-positive clinical isolates, (**B**) Gram-negative clinical isolates, and (**C**) Gram-negative AR isolates. VME, very major error; ME, major error; mE, minor error. %, error rate percentages for VME, ME, and mE of each drug–bug combination as described in the method section. CLI, clindamycin; CPT, ceftaroline; ERY, erythromycin; OXA, oxacillin; PEN, penicillin; SXT, trimethoprim/sulfamethoxazole; VAN, vancomycin; AMC, amoxicillin/clavulanate; AMK, amikacin; ATM, aztreonam; CAZ, ceftazidime; CFZ, cefazolin; CIP, ciprofloxacin; CRO, ceftriaxone; CZA, ceftazidime/avibactam; ETP, ertapenem; FEP, cefepime; FOX, cefoxitin; GEN, gentamicin; LVX, levofloxacin; MEM, meropenem; MIN, minocycline; SAM, ampicillin/sulbactam; SXT, trimethoprim/sulfamethoxazole; TOB, tobramycin; TZP, piperacillin/tazobactam.

Across 5124 tested drug–bug combinations, 300 categorical discrepancies were identified, including 55 very major errors (VMEs; 1.1%), 42 major errors (MEs; 0.8%), and 203 minor errors (mEs; 4.0%) (Supplementary Table S3). Among the 937 combinations with GP clinical isolates, 6 VMEs (0.6%), 10 MEs (1.1%), and 11 mEs (1.2%) were observed. *Staphylococcus* spp. accounted for most VMEs (4/6) with clindamycin (CLI), with the remainder involving penicillin (PEN) and trimethoprim/sulfamethoxazole (SXT). They also contributed 4 of 10 MEs with SXT and 3 of 11 mEs with ceftaroline (CPT). *Enterococcus* spp. were responsible for 3 of 10 MEs with vancomycin (VAN), 2 MEs with ERY, and 6 mEs with ERY (Figure 2A and Supplementary Table S3).

For GN clinical isolates, 30 VMEs (0.9%), 28 MEs (0.9%), and 130 mEs (4.0%) were identified among 3249 drug–bug combinations (Supplementary Table S3). Most discrepancies occurred in Enterobacterales, which accounted for 27 of 30 VMEs (90%), 21 of 28 MEs (75%), and 119 of 130 mEs (91.5%), mainly involving ATM, ceftazidime (CAZ), CFZ, ciprofloxacin (CIP), ceftriaxone (CRO), FEP, FOX, GEN, levofloxacin (LVX), MIN, SAM, and TZP (Figure 2B and Supplementary Table S3).

Among the GN AR Bank reference isolates, 19 VMEs (2.0%), 4 MEs (0.4%), and 62 mEs (6.7%) were identified across 938 drug–bug combinations (Supplementary Table S3). Enterobacterales again accounted for the majority of VMEs (13/19, 68.4%) and a substantial portion of mEs (24/62, 38.7%). *P. aeruginosa* contributed 3 of 4 MEs (75%) with ceftazidime/avibactam (CZA) and most mEs (25/62, 40.3%), including discrepancies with AMK, ATM, CIP, FEP, LVX, MEM, TOB, and TZP (Figure 2C and Supplementary Table S3). In addition, *Acinetobacter baumannii* accounted for 13 mEs (13/62, 21.0%), including 1 with AMK, 8 with MIN, and 4 with SAM. Taken together, most discrepancies were concentrated in Enterobacterales and *P. aeruginosa*, consistent across both clinical and reference isolates.

**Figure 3:**
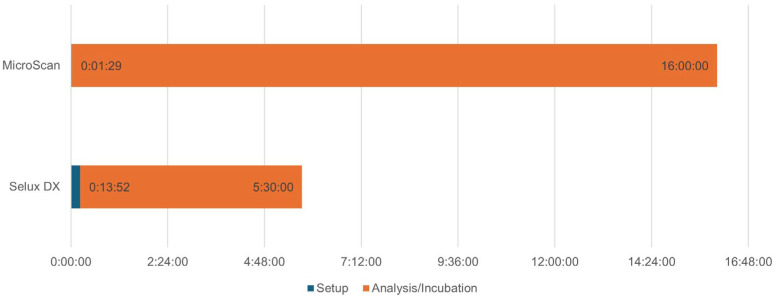
Turnaround time comparison between the Selux DX Analyzer (excluding the required McFarland turbidity setup time) and the MicroScan system.

In terms of turnaround time, the Selux DX Analyzer exhibited a Time-to-result (TTR) ranging from 5:05:17 (hour:min:sec) to 8:28:41, with a standard deviation (SD) of 0:29:55. Longer TTRs were primarily observed for *P. aeruginosa,* which grows more slowly than other GN isolates tested. When *P. aeruginosa* was excluded, the TTR range narrowed to 5:05:17 to 7:01:34, with a reduced SD of 0:07:48. Notably, the Selux DX system demonstrated a significantly shorter TTR, with an average of 5.5 h—substantially faster than the MicroScan system, which required an average of 16 h (Figure 3). However, the average setup time for processing a single tray containing up to four AST panels using the Selux DX Inoculator was 00:13:52 ± 00:03:04 (range: 00:03:45–00:17:44), excluding the time required to prepare the initial colony suspension to the required McFarland turbidity. In contrast, the MicroScan system requires manual sample setup and takes approximately 1 min 29 s per isolate [16].

## 3. Discussion

In this single-center study, we compared the AST performance of the Selux DX and MicroScan systems using a diverse collection of GP and GN bacterial isolates. Our findings suggest that the Selux DX system is comparable to the MicroScan platform in terms of CA, while offering a substantial reduction in turnaround time. Although it requires a longer sample setup time, the Selux DX system presents a key advantage for enhancing antimicrobial stewardship efforts by enabling faster reporting of susceptibility results. Consistent with the findings reported by Baker et al., the Selux DX system achieved ≥90% CA for most drug–bug combinations, supporting its reliability and clinical applicability in routine clinical microbiology laboratory workflows [17]. Notably, certain antibiotics, such as CFZ and MIN, demonstrated lower CA in our evaluation. For CFZ, the discrepancies were concentrated in Enterobacterales, particularly *Proteus mirabilis*, where intrinsic resistance and complex cephalosporin breakpoint interpretations may have contributed to discordant results [18]. For MIN, discordances were primarily associated with Enterobacterales (particularly, *K. pneumoniae*) and *A. baumannii*. Prior studies have noted variability in MIN MIC testing, especially for nonfermenters, which may reflect differences in drug uptake, efflux mechanisms, and challenges in standardizing in vitro testing for MIN [19,20,21]. These biological and methodological factors likely contributed to the lower CA observed for these agents in our dataset.

According to CLSI and FDA recommendations, acceptable performance thresholds include VME and ME rates ≤ 3% and mE rates ≤ 10% [22,23]. In our study, most combinations met these criteria; however, several exceeded the limits. For Gram-positive clinical isolates, CLI against *Staphylococcus* spp. showed a VME rate of 17.4%, while VAN against *Enterococcus* spp. exhibited an ME rate of 12% and an mE rate of 3.8%. For Gram-negative clinical isolates, elevated error rates were observed with CFZ against Enterobacterales (mE 26.6%), MIN against Enterobacterales (VME 23%, mE 7.1%), and SAM against Enterobacterales (mE 10.9%). Among the AR Bank reference isolates, high-than-threshold error rates included MIN against *A. baumannii* (mE 32%), FOX against Enterobacterales (VME 11.1%, mE 12.5%), GEN against Enterobacteralis (VME 16.7%), and MEM against Enterobacterales (mE 18.8%). These exceedances likely reflect the smaller sample size and more limited bacterial diversity tested in our single-center study compared with the broader dataset analyzed by Baker et al. [17].

While we evaluated the CA of the Selux DX system against reference methods such as the MicroScan system for clinical isolates and the broth microdilution method used by AR Bank, we were unable to assess essential agreement (EA), which is defined as MIC values falling within ±1 doubling dilution between the test and reference methods. In this study, most susceptibility results were reported only as “greater than” or “less than” a specific MIC, limiting the number of isolates eligible for EA analysis. The inability to assess EA therefore precluded a comprehensive comparison of MIC concordance among the different AST methods, and limits the depth of our performance evaluation. EA provides a measure of how closely MIC values generated by a test method align with those of a reference method, beyond categorical agreement. This is clinically important because small MIC shifts within a category may still influence antibiotic selection, particularly for agents with narrow therapeutic windows or when dosing strategies are employed. Without EA analysis, it remains uncertain whether the Selux DX system consistently reproduces MIC values near clinical breakpoints, where even a one-dilution difference could change patient management decisions. Moreover, the absence of EA prevents identification of systematic MIC shifts that might not alter categorical results but could compromise the accuracy of reported MIC values. It also limits the ability to detect error patterns, such as a consistent bias toward higher or lower MICs, that would otherwise be revealed through EA analysis. Relying solely on CA introduces interpretive limitations, particularly regarding how MIC values are translated into categorical results. For example, while both Selux DX and MicroScan used systemic CLSI or FDA breakpoints, MicroScan, in alignment with CLSI, also considers alternative breakpoints for urinary isolates in uncomplicated urinary tract infections (UTIs). In contrast, the Selux DX system, following FDA standards, does not incorporate this distinction. Within our dataset, this difference was reflected in a single *E. coli* urinary isolate, which Selux DX categorized as resistant, but MicroScan classified as susceptible. Future studies should address this deficiency by incorporating broth microdilution as the reference method, ensuring that sufficient MIC data are available to allow for robust EA analysis. This approach will be critical to fully defining the accuracy, reproducibility, and clinical reliability of the Selux DX system.

A key advantage of the Selux DX system is its significantly shorter TTR compared to the MicroScan system. With an average AST TTR of 5.5 h, the Selux DX system markedly reduced the time required to generate actionable susceptibility data, compared with the 16 h average for MicroScan. This accelerated reporting has important clinical implications, including earlier optimization of antibiotic therapy and timely de-escalation from broad-spectrum empiric regimens to targeted antibiotics. Such improvements may enhance patient outcomes, reduce selective pressure for resistance, and minimize unnecessary exposure to potentially toxic agents. However, fully automated AST systems can still encounter occasional errors and downtime due to instrument malfunctions [24]. In such cases, delayed reporting underscores the importance of maintaining manual testing approaches as a reliable backup for clinical laboratories.

The Selux DX system is also designed with future adaptability in mind. Its high-throughput 384-well plate format, containing 15 antibiotics for GP and 24 for GN panels, facilitates the addition of newly approved antibiotics without requiring major reconfiguration. This modular design reduces the need for reflex testing and allows clinical laboratories to quickly incorporate novel antimicrobials into routine AST workflows. Nonetheless, challenges remain with respect to instrument footprint, workflow integration, and cost, which may impact implementation across laboratories of varying size and resources.

From a workflow perspective, institutions that do not operate on a 24/7 schedule may be unable to fully leverage the system’s rapid TTR. Without continuous staffing, AST results may not be reviewed or communicated to clinicians in real time, limiting clinical impact. Furthermore, the utility of rapid AST platforms is closely linked to the antimicrobial stewardship programs (ASPs) infrastructure [25]. Facilities with limited ASP resources or intermittent stewardship coverage may struggle to act promptly on rapid susceptibility results, thereby diminishing the benefits of accelerated reporting.

Another important consideration is the cost-effectiveness of implementing the Selux DX system in routine clinical practice. While the system offers improved turnaround times and potential clinical advantages, formal economic analyses are needed to determine whether these benefits justify the costs, particularly for laboratories already using established AST platforms like MicroScan [8,26,27]. Studies assessing the financial implications, including potential reductions in hospital length of stay, antibiotic costs, and downstream healthcare savings, will be critical in guiding adoption.

Additionally, discrepancies observed between the two platforms for clinical isolates could not be resolved through further testing, as these isolates were not preserved. This limitation precluded confirmation with a third independent method, such as broth microdilution. Notably, we observed a greater number of drugs with <90% CA among the GN AR Bank isolates compared with clinical isolates. Given that AR Bank isolates have well-established susceptibility profiles, this raises concerns. CLSI criteria for acceptable method validation require ≥90% CA with VME and ME rates ≤ 3% and mE rates ≤ 10%. Our findings may reflect challenges in testing highly resistant or diverse reference strains, suggesting that the Selux DX system’s performance for certain drug–bug combinations warrants further evaluation in larger multicenter studies.

Lastly, as a single-center evaluation, our study reflects the patient population, antimicrobial resistance patterns, and laboratory practices unique to our institution. These factors may limit generalization. Broader validation through multicenter studies incorporating diverse geographic regions, expanded isolate collections, and standardized reference testing will be necessary to fully assess the performance and applicability of the Selux DX system.

## 4. Materials and Methods

### 4.1. Bacterial Isolates

Two sets of bacterial isolates were collected and analyzed in this study: (i) Clinical Isolates—A total of 332 clinical isolates, including 109 GP and 223 GN organisms, were randomly obtained from the Clinical Microbiology Laboratory at the University of Texas Medical Branch at Galveston, Texas, from March to May 2024. Sources of samples are summarized in Table 2. These isolates were recovered from patient specimens as part of routine diagnostic testing. Antimicrobial susceptibility data for these isolates were generated using the MicroScan WalkAway Plus Microbiology System with NM56 and PM38 Panels (Beckman Coulter, Brea, CA, USA), which serves as the current Standard of Care (SOC) method. (ii) Reference Isolates—To ensure a comprehensive assessment of the Selux DX system across a broad range of antimicrobial resistance profiles, 97 GN reference isolates from AR Bank. These isolates represent clinically significant antimicrobial resistance mechanisms and serve as benchmarks for AST performance evaluation. In addition, 12 quality control (QC) strains recommended by the manufacturer, comprising 6 GP and 6 GN organisms, were included in testing to verify assay reproducibility and performance consistency of the Selux DX system (Selux Diagnostics Inc., Charlestown, MA, USA).

### 4.2. Selux Next-Generation Phenotyping AST System

All bacterial isolates were processed according to the manufacturer’s instructions. Briefly, isolates were subcultured on Remel 5% sheep blood agar (Thermo Fisher Scientific, Waltham, MA, USA) and incubated for 18–24 h. Colonies were then suspended in 2.5 mL of saline to achieve a turbidity of a 0.4–0.6 McFarland standard. Each standardized suspension was paired with the corresponding AST panel: the GP panel contained 15 antibiotics, and the GN panel contained 24 antibiotics.

The prepared bacterial suspensions were automatically diluted in cation-adjusted Mueller-Hinton broth and inoculated into the AST panels using the Selux DX Inoculator (Selux Diagnostics Inc., Charlestown, MA, USA). The inoculated panels were subsequently loaded into the Selux DX Analyzer (Selux Diagnostics Inc., Charlestown, MA, USA), where incubation and real-time analysis occurred. Once bacterial identification was entered into the system’s controlling software, the Selux DX Analyzer determined the MIC for each antibiotic and applied the appropriate interpretive criteria based on CLSI or FDA breakpoints. TTR, defined as the duration from panel setup to final result, was recorded directly from the Selux DX system.

### 4.3. Data Analysis

The MIC values and interpretive categorizations obtained using the Selux DX system were compared to those generated by the SOC MicroScan system (Beckman Coulter Inc., Franklin Lakes, NJ, USA) for clinical isolates, and to the established susceptibility profiles for AR Bank reference isolates. CA, which refers to the concordance of susceptibility interpretations (susceptible, intermediate, susceptible-dose-dependent [SDD], or resistant) regardless of the exact MIC value, was assessed. CA percentages were calculated for each drug–organism combination. Both MicroScan and Selux Dx systems follow the most current CLSI M100/FDA breakpoints in effect during the isolate collection period, which spanned from March to May 2024.

VMEs, MEs, and mEs were classified according to standard CLSI definitions [28]. In this study, a VME was recorded when an isolate categorized as resistant by the MicroScan method or AR Bank reference data was interpreted as susceptible by the Selux DX system. An ME was recorded when an isolate categorized as susceptible by the reference method was reported as resistant by Selux DX. A mE occurred when one method reported an intermediate or SDD result, while the other reported either susceptible or resistant. All discrepancies were tallied for each drug–organism combination.

Very Major Error Rate (%) = (Number of VMEs/Number of resistant isolates by the reference method) × 100

Major Error Rate (%) = (Number of MEs/Number of susceptible isolates by the reference method) × 100

Minor Error Rate (%) = (Number of mEs/Total number of isolates tested) × 100

## 5. Conclusions

Rapid phenotypic AST platforms like the Selux DX system, when integrated with well-structured ASPs, hold considerable promise for transforming the management of bacterial infections. By delivering timely and accurate susceptibility results, these systems can support more precise antibiotic selection, improve clinical outcomes, and help mitigate the global threat of antimicrobial resistance. Future multicenter studies with larger and more diverse isolate collections, complemented by cost-effectiveness analyses and real-world implementation studies, will be essential to further establish the clinical utility and broader adoption potential of the Selux DX system across varied healthcare environments.

## Figures and Tables

**Table 1:** List of Clinical and AR Bank Organisms.

	Clinical Isolates		AR Bank Isolates	
Organisms	Number	Percentage	Number	Percentage
*Enterococcus faecalis*	24	22.0%	-	-
*Enterococcus faecium*	2	1.8%	-	-
*Staphylococcus aureus*	60	55.0%	-	-
*Staphylococcus capitis*	2	1.8%	-	-
*Staphylococcus epidermidis*	15	13.8%	-	-
*Staphylococcus haemolyticus*	1	0.9%	-	-
*Staphylococcus lugdunensis*	5	4.6%	**-**	**-**
**Gram-Positive Total**	**109**		**-**	**-**
*Acinetobacter baumannii*	7	3.1%	25	25.8%
*Citrobacter freundii*	1	0.4%	1	1.0%
*Citrobacter koseri*	5	2.2%	4	4.1%
*Enterobacter cloacae*	4	1.8%	1	1.0%
*Escherichia coli*	68	30.5%	5	5.2%
*Klebsiella aerogenes*	2	0.9%	1	1.0%
*Klebsiella oxytoca*	3	1.3%	1	1.0%
*Klebsiella pneumoniae*	38	17.0%	16	16.5%
*Klebsiella variicola*	3	1.3%	-	-
*Morganella morganii*	2	0.9%	1	1.0%
*Proteus mirabilis*	45	20.2%	3	3.1%
*Proteus vulgaris*	4	1.8%	-	-
*Pseudomonas aeruginosa*	32	14.3%	33	34.0%
*Serratia marcescens*	9	4.0%	6	6.2%
**Gram-Negative Total**	**223**		**97**	

**Table 2:** Sources of clinical isolates.

Source	Gram-Positive	Gram-Negative
Abdomen fluid	0	2
Blood	10	8
Bone	4	3
Cerebrospinal fluid	1	1
Sputum	3	11
Stool	0	1
Swab	43	34
Tissue	6	5
Urine	23	141
Wound	19	17
Total	109	223

## Data Availability

The original contributions presented in the study are included in the article. Further inquiries can be directed to the corresponding author.

## Supplementary Materials

The following supporting information can be downloaded at: https://www.mdpi.com/article/10.3390/antibiotics14100962/s1, Table S1: Categorical Agreement (CA) of Gram-Positive Clinical Isolates; Table S2: Categorical Agreement (CA) of Gram-Negative Clinical and AR Bank Isolates; Table S3: List of Categorical Discrepancies.
