# Supplementary material for: Evaluation of a Novel Rapid Phenotypic Antimicrobial Susceptibility Testing System
Source: Antibiotics (Basel). 2025 Sep 25;14(10):962. doi: 10.3390/antibiotics14100962 (PMC12562109; doi:10.3390/antibiotics14100962)
Supplement: Supplementary file 1 [file antibiotics-14-00962-s001.zip › antibiotics-3863202-supplementary.pdf]

antibiotics

MDPI

Supplementary Tables:

Supplementary Table S1. Categorical Agreement (CA) of Gram-Positive Clinical Isolates.

Abbreviation	Gram-Positive Drug	Clinical Isolates CA%
AMP	Ampicillin	100% (26/26)
CLI	Clindamycin	94.1% (64/68)
CPT	Ceftaroline	95.0% (57/60)
DAP	Daptomycin	100% (84/84)
ERY	Erythromycin	89.5% (77/86)
FOX	Cefoxitin Screen	100% (65/65)
LNZ	Linezolid	100% (109/109)
LVX	Levofloxacin	100% (86/86)
OXA	Oxacillin	98.8% (82/83)
PEN	Penicillin	98.8% (80/81)
SXT	Trimethoprim/ Sulfamethoxazole	93.8% (75/80)
VAN	Vancomycin	96.3% (105/109)

Supplementary Table S2. Categorical Agreement (CA) of Gram-Negative Clinical and AR Bank Isolates.

Abbreviation	Gram-Negative Drug	Clinical Isolates CA%	AR Bank Isolates CA%	Overall CA%
AMC	Amoxicillin/ Clavulanate	97.0% (160/165)	88.0% (22/25)	95.8% (182/190)
AMK	Amikacin	97.4% (38/39)	88.0% (44/50)	92.1% (82/89)
AMP	Ampicillin	-	100% (8/8)	100% (8/8)
ATM	Aztreonam	89.2% (83/93)	84.4% (27/32)	88.0% (110/125)
CAZ	Ceftazidime	94.1% (177/188)	93.7% (59/63)	94.0% (236/251)
CFZ	Cefazolin	69.0% (109/158)	100% (24/24)	72.5% (132/182)
CIP	Ciprofloxacin	95.4% (206/216)	95.8% (68/71)	95.5% (274/287)
CRO	Ceftriaxone	98.7% (152/154)	96.3% (26/27)	95.0% (172/181)
CZA	Ceftazidime/Avibactam	99.0% (205/207)	94.6% (53/56)	98.1% (258/263)
ETP	Ertapenem	100% (183/183)	94.4% (34/36)	99.1% (217/219)
FEP	Cefepime	94.4% (201/213)	89.4% (59/66)	93.2% (260/279)
FOX	Cefoxitin	93.8% (165/176)	78.1% (25/32)	91.3% (190/208)
GEN	Gentamicin	96.7% (208/215)	87.2% (34/39)	95.3% (242/254)
IMP	Imipenem	100% (7/7)	100% (25/25)	100% (32/32)
LMR	Imipenem/Relebactam	-	100% (11/11)	100% (11/11)
LVX	Levofloxacin	94.4% (203/215)	97.2% (69/71)	95.1% (272/286)

MEM	Meropenem	98.6% (219/222)	86.9% (73/84)	95.4% (292/306)
MEV	Meropenem/ Vaborbactam	100% (175/175)	100% (5/5)	100% (180/180)
MIN	Minocycline	90.0% (108/120)	64.3% (18/28)	85.1% (126/148)
SAM	Ampicillin/Sulbactam	85.1% (148/174)	90.2% (46/51)	86.2% (194/225)
SXT	Trimethoprim/ Sulfamethoxazole	97.3% (72/74)	100% (27/27)	98.0% (99/101)
TOB	Tobramycin	96.9% (31/32)	90.6% (29/32)	93.8% (60/64)
TZP	Piperacillin/ Tazobactam	94.4% (203/215)	88.7% (63/71)	93.0% (266/286)

Supplementary Table S3. List of Categorical Discrepancies.

**Gram-Positive Clinical isolates**											
		**S**[1]	**I/SDD**[1]	**R**[1]	**T**[1]	**VME**[1]		**ME**[1]		**mE**[1]	
**Abbreviation of antibiotics**[2]	**Organisms**[5]	#	#	#	#	#	%[4]	#	%[4]	#	%[4]
AMP	*Enterococcus* spp.: *EFS, EFM*	25	-	1	26	-	-	-	-	-	-
CLI	*Staphylococcus* spp.: *SA, SC, SE, SL, SH*	49	-	19	68	4	17.4	-	-	-	-
CPT	*Staphylococcus* spp.: *SA*	57	3	-	60	-	-	-	-	3	5
DAP	*Staphylococcus* spp.: *SA*	60	-	-	60	-	-	-	-	-	-
DAP	*Enterococcus* spp.: *EFS*	24	-	-	24	-	-	-	-	-	-
ERY	*Staphylococcus* spp.: *SA, SC, SE, SL, SH*	29	-	39	68	-	-	-	-	1	1.5
ERY	*Enterococcus* spp.: *EFS, EFM*	1	10	7	18	-	-	2	33.3	6	33.3
FOX	*Staphylococcus* spp.: *SA, SL*	37	-	28	65	-	-	-	-	-	-
LNZ	*Staphylococcus* spp.: *SA, SC, SE, SL, SH*	83	-	-	83	-	-	-	-	-	-
LNZ	*Enterococcus* spp.: *EFS, EFM*	26	-	-	26	-	-	-	-	-	-
LVX	*Staphylococcus* spp.: *SA*	37	-	23	60	-	-	-	-	-	-
LVX	*Enterococcus* spp.: *EFS, EFM*	19	1	6	26	-	-	-	-	-	-
OXA	*Staphylococcus* spp.: *SA, SC, SE, SL, SH*	42	-	41	83	-	-	1	2.3	-	-

PEN	*Staphylococcus* spp.: *SA*	10	-	50	60	1	2.0	-	-	-	-
PEN	*Enterococcus* spp.: *EFS, EFM*	21	-	-	21	-	-	-	-	-	-
SXT	*Staphylococcus* spp.: *SA, SC, SE, SL, SH*	62	-	18	80	1	5.3	4	6.1	-	-
VAN	*Staphylococcus* spp.: *SA, SC, SE, SL, SH*	83	-	-	83	-	-	-	-	-	-
VAN	*Enterococcus* spp.: *EFS, EFM*	22	-	4	26	-	-	3	12	1	3.8
	Total	687	14	236	937	6		10		11	

Gram-Negative Clinical Isolates											
		**S**[1]	**I/SDD**[1]	**R**[1]	**T**[1]	**VME**[1]		**ME**[1]		**mE**[1]	
**Abbreviation of antibiotics**[3]	**Organisms**[6]	#	#	#	#	#	%[4]	#	%[4]	#	%[4]
AMC	Enterobacterales: *CK, EC, KO, KP, KV, PM, PV*	149	11	5	165	-	-	-	-	5	3.0
AMK	*Pseudomonas aeruginosa*	32	-	-	32	-	-	-	-	1	3.1
AMK	*Acinetobacter baumannii*	7	-	-	7	-	-	-	-	-	-
ATM	Enterobacterales: *EC*	57	5	5	67	1	11.1	-	-	5	7.5
ATM	*Pseudomonas aeruginosa*	22	2	2	26	1	50	-	-	3	11.5
CAZ	Enterobacterales: *CF, CK, ECC, EC, KA, KO, KP, KV, PM, PV, SM*	166	4	11	181	3	18.8	-	-	6	3.3
CAZ	*Acinetobacter baumannii*	5	-	2	7	-	-	2	28.6	-	-
CFZ	Enterobacterales: *CK,EC, KP, KV, PM*	82	47	29	158	2	6.1	5	4.4	42	26.6
CZA	Enterobacterales: *CF, CK, ECC, EC, KA, KO, KP, KV, MM, PM, PV, SM*	176	-	-	176	-	-	-	-	-	-
CZA	*Pseudomonas aeruginosa*	31	-	1	32	1	100	1	3.2	-	-
CIP	Enterobacterales: *CF, CK, ECC, EC, KA, KO, KP, KV, MM, PM, PV, SM*	155	5	23	183	3	10	-	-	7	3.8
CIP	*Pseudomonas aeruginosa*	29	-	3	32	-	-	-	-	-	-

CRO	Enterobacterales: *CF, CK, EC, KA, KO, KP, KV, PM*	145	2	17	164	2	10.5	-	-	-	-
ETP	Enterobacterales: *CF, CK, ECC, EC, KA, KO, KP, KV, MM, PM, PV, SM*	183	-	-	183	-	-	-	-	-	-
FEP	Enterobacterales: *CF, CK, ECC, EC, KA, KO, KP, KV, MM, PM, PV, SM*	165	6	12	183	3	17.6	2	1.2	6	3.3
FEP	*Pseudomonas aeruginosa*	28	-	2	30	-	-	-	-	1	3.3
FOX	Enterobacterales: *CK, EC, KO, KP, KV, MM, PM, PV, SM*	159	4	13	176	1	10	1	0.6	9	5.1
GEN	Enterobacterales: *CF, CK, ECC, EC, KA, KO, KP, KV, MM, PM, PV, SM*	178	1	4	183	3	37.5	-	-	2	1.1
GEN	*Pseudomonas aeruginosa*	32	-	-	32	1	100	-	-	1	3.1
IMP	*Acinetobacter baumannii*	5	-	2	7	-	-	-	-	-	-
LVX	Enterobacterales: *CF, CK, ECC, EC, KA, KO, KP, KV, MM, PM, PV, SM*	151	9	23	183	2	8.0	-	-	9	4.9
LVX	*Pseudomonas aeruginosa*	25	4	3	32	-	-	-	-	1	3.1
MEM	Enterobacterales: *CF, CK, ECC, EC, KA, KO, KP, KV, MM, PM, PV, SM*	186	1	3	190	-	-	1	0.5	1	0.5
MEM	*Pseudomonas aeruginosa*	31	1	-	32	-	-	-	-	1	3.1
MEV	Enterobacterales: *CF, CK, ECC, EC, KA, KO, KP, KV, MM, PM, PV, SM*	175	-	-	175	-	-	-	-	-	-
MIN	Enterobacterales: *EC, KA, KO, KP, KV*	101	4	8	113	3	23	-	-	8	7.1
MIN	*Acinetobacter baumannii*	6	1	-	7	-	-	-	-	1	14.3
SAM	Enterobacterales: *CK, EC, KO, KP, KV, MM, PM, PV*	117	21	27	165	1	5.0	4	3.2	18	10.9
SAM	*Acinetobacter baumannii*	4	1	2	7	-	-	2	28.6	1	14.3

SXT	Enterobacterales: *CF, CK, ECC, EC, KA, KO, KP, KV*	49	-	25	74	1	4.0	1	2.0	-	-
TOB	*Pseudomonas aeruginosa*	32	-	-	32	-	-	-	-	1	3.1
TZP	Enterobacterales: *CF, CK, ECC, EC, KA, KO, KP, KV, MM, PM, PV, SM*	173	1	9	183	2	50	7	3.9	1	0.5
TZP	*Pseudomonas aeruginosa*	29	-	3	32	-	-	2	6.5	-	-
Total		2885	130	234	3249	30		28		130	

**Gram-Negative AR Bank Isolates**											
		**S**[1]	**I/SDD**[1]	**R**[1]	**T**[1]	**VME**[1]		**ME**[1]		**mE**[1]	
**Abbreviation of antibiotics**[3]	**Organisms**[6]	#	#	#	#	#	%[4]	#	%[4]	#	%[4]
AMC	Enterobacterales: *EC, KO, KP, PM*	2	1	22	25	2	8.0	-	-	1	4.0
AMK	*Pseudomonas aeruginosa*	21	3	9	33	1	12.5	-	-	4	12.1
AMK	*Acinetobacter baumannii*	1	2	14	17	-	-	-	-	1	5.9
AMP	Enterobacterales: *EC, PM*	-	-	8	8	-	-	-	-	-	-
ATM	*Pseudomonas aeruginosa*	12	4	11	27	-	-	-	-	5	18.5
ATM	Enterobacterales: *EC*	1	-	4	5	-	-	-	-	-	-
CAZ	Enterobacterales: *CF, ECC, EC, KA, KO, KP, PM, SM*	9	1	27	37	1	3.7	-	-	2	5.4
CAZ	*Acinetobacter baumannii*	1	-	24	25	1	4.0	-	-	-	-
CFZ	Enterobacterales: *EC, KP, PM*	-	-	24	24	-	-	-	-	-	-
CIP	Enterobacterales: *CF, ECC, EC, KA, KO, KP, MM, PM, SM*	13	-	26	39	1	3.7	-	-	-	-
CIP	*Pseudomonas aeruginosa*	7	-	25	32	1	3.8	-	-	1	3.1
CRO	Enterobacterales: *CF, EC, KA, KO, KP, PM*	1	-	26	27	-	-	-	-	1	3.7
CZA	Enterobacterales: *CF, ECC, EC, KA, KO, KP, MM, PM, SM*	13	-	12	25	-	-	-	-	-	-
CZA	*Pseudomonas aeruginosa*	19	-	12	31	-	-	3	13.6	-	-

ETP	Enterobacterales: *ECC, EC, KA, KO, KP, MM, PM, SM*	3	-	33	36	1	2.9	-	-	1	2.8
FEP	Enterobacterales: *CF, ECC, EC, KA, KO, KP, MM, PM, SM*	10	1	27	38	-	-	-	-	1	2.6
FEP	*Pseudomonas aeruginosa*	13	5	10	28	1	9.1	-	-	5	17.9
FOX	Enterobacterales: *EC, KO, KP, MM, PM, SM*	5	2	25	32	3	11.1	-	-	4	12.5
GEN	Enterobacterales: *CF, ECC, EC, KA, KO, KP, MM, PM, SM*	22	2	15	39	3	16.7	-	-	2	5.1
IMP	*Acinetobacter baumannii*	-	-	25	25	-	-	-	-	-	-
IMR	Enterobacterales: *EC, KP*	1	-	2	3	-	-	-	-	-	-
IMR	*Pseudomonas aeruginosa*	6		2	8	-	-	-	-	-	-
LVX	Enterobacterales: *CF, ECC, EC, KA, KO, KP, MM, PM, SM*	11	1	27	39	-	-	-	-	1	2.6
LVX	*Pseudomonas aeruginosa*	5	2	25	32	-	-	-	-	1	3.1
MEM	Enterobacterales: *ECC, EC, KA, KO, KP, MM, PM*	4	9	19	32	1	4	-	-	6	18.8
MEM	*Pseudomonas aeruginosa*	10	8	14	32	1	6.7	-	-	3	9.4
MEM	*Acinetobacter baumannii*	-	-	25	25	-	-	-	-	-	-
MEV	Enterobacterales: *ECC, KP, SM*	5	-	-	5	-	-	-	-	-	-
MIN	Enterobacterales: *EC, KP*	3	-	-	3	1	100	-	-	-	-
MIN	*Acinetobacter baumannii*	8	1	16	25	-	-	1	12.5	8	32
SAM	Enterobacterales: *EC, KO, KP, MM, PM*	-	1	25	26	-	-	-	-	1	3.8
SAM	*Acinetobacter baumannii*	-	2	23	25	-	-	-	-	4	16
SXT	Enterobacterales: *CF, ECC, EC, KA, KO, KP*	4	-	23	27	-	-	-	-	-	-
TOB	*Pseudomonas aeruginosa*	12	4	16	32	-	-	-	-	3	9.4

TZP	Enterobacterales: *CF, ECC, EC, KA, KO, KP, MM, PM, SM*	7	3	29	39	-	-	-	-	4	10.3
TZP	*Pseudomonas aeruginosa*	19	2	11	32	1	8.3	-	-	3	9.4
	Total	248	54	636	938	19		4		62	

**Summary Data**	**T**[1]	**VME**[1]	**ME**[1]	**mE**[1]
	5124	55	42	203

[1]VME, very major error; ME, major error; mE, minor error; S, susceptible isolates; I/SDD, intermediate/ susceptible-dose dependent isolates; R, resistant isolates; T, total isolates.

[2]AMP, ampicillin; CLI, clindamycin; CPT, ceftaroline; DAP, daptomycin; ERY, erythromycin; FOX, cefoxitin screen; LNZ, linezolid; LVX, levofloxacin; OXA, oxacillin; PEN, penicillin; SXT, trimethoprim/sulfamethoxazole; VAN, vancomycin.

[3]AMC, amoxicillin/clavulanate; AMK, amikacin; AMP, ampicillin; ATM, aztreonam; CAZ, ceftazidime; CFZ, cefazolin; CIP, ciprofloxacin; CRO, ceftriaxone; CZA, ceftazidime/avibactam; ETP, ertapenem; FEP, cefepime; FOX, cefoxitin; GEN, gentamicin; IMP, imipenem; IMR, imipenem/relebactam; LVX, levofloxacin; MEM, meropenem; MEV, meropenem/vaborbactam; MIN, minocycline; SAM, ampicillin/sulbactam; SXT, trimethoprim/sulfamethoxazole; TOB, tobramycin; TZP, piperacillin/tazobactam

[4]%, error rate percentages for VME, ME, and mE of each drug-bug combination as described in the method section

[5]EFS, *Enterococcus faecalis*; EFM, *Enterococcus faecium*; SA, *Staphylococcus aureus*; SC, *Staphylococcus capitis*; SE, *Staphylococcus epidermidis*; SL, *Staphylococcus lugdunensis*; SH, *Staphylococcus haemolyticus*

[6]The Enterobacterales group includes species: CF, *Citrobacter freundii* complex; CK, *Citrobacter koseri*; ECC, *Enterobacter cloacae* complex; EC, *Escherichia coli*; KA, *Klebsiella aerogenes*; KO, *Klebsiella oxytoca*; KP, *Klebsiella pneumoniae*; KV, *Klebsiella variicola*; MM, *Morganella morganii*; PM, *Proteus mirabilis*; PV, *Proteus vulgaris*; SM, *Serratia marcescens*.
